# Impact of Bone Augmentation of Facial Bone Defect around Osseointegrated Implant: A Three Dimensional Finite Element Analysis

**DOI:** 10.3390/dj9100114

**Published:** 2021-10-03

**Authors:** Malik Hudieb, Mustafa AlKhader, Salah Mortaja, Mohammad Abusamak, Noriyuki Wakabayashi, Shohei Kasugai

**Affiliations:** 1 Section of Periodontology, Department of Preventive Dentistry, Jordan University of Science and Technology, Irbid 22110, Jordan; 2 Department of Oral Medicine and Oral Surgery, Jordan University of Science and Technology, Irbid 22110, Jordan; mmalkhader@just.edu.jo; 3 The Chris O’Brien Lifehouse, Sydney, NSW 2050, Australia; smortaja90@gmail.com; 4 Faculty of Dentistry, McGill University, Montréal, QC H3A 1G1, Canada; mohammad.abusamak@mail.mcgill.ca; 5 Removable Partial Prosthodontics Department, Graduate School of Medical and Dental Sciences, Tokyo Medical and Dental University, Tokyo 113-8510, Japan; wakabayashi.rpro@tmd.ac.jp; 6 Oral Implantology and Regenerative Dental Medicine Department, Tokyo Medical and Dental University, Tokyo 113-8510, Japan; kas.mfc@tmd.ac.jp

**Keywords:** fenestration, dental implant, esthetic zone, finite element analysis, strain

## Abstract

(1) Background: When dental implants are placed at the esthetic zone, facial bone fenestration might be expected. This study aimed to evaluate the biomechanical effect of bone augmentation around implants with facial bone fenestration defects using the finite element method. (2) Methods: An anterior maxillary region model with facial concavity was constructed with a threaded implant inserted following the root direction, resulting in apical threads exposure to represent the fenestration model. Several bone coverage levels were simulated by gradually shifting the deepest concavity point buccally, mimicking bone augmentation surgeries with different bone fill results. Oblique forces were applied, and analysis was performed. (3) Results: Peak compressive stress magnitude and distribution varied according to the level of exposure and facial concavity depth. The fenestration model demonstrated a slightly lower peak peri-implant bone stress, smaller implant displacement, and smaller bone volume with strain levels above 200 µ strain. A gradual increase in compressive stress, implant displacement, and bone volume exhibited strain level above 200 µ strain was observed with the increased bone fill level of the facial bone fenestration. (4) Conclusions: Exposure of implants apical threads at the maxillary anterior region does not significantly affect the peri-implant stress and strain results. However, increasing the buccolingual width and eliminating the buccal concavity might increase the peri-implant bone volume exhibited favorable loading levels.

## 1. Introduction

Oral implants have been successfully used to restore function and esthetics of missing teeth in partially and fully edentulous patients. Mostly, the treatment comprises first of surgical placement of the root-form titanium screw followed after the healing period by the prosthetic loading. In fact, several treatment protocols have been established regarding implant placement and loading timing, including immediate, early, and late protocols [1]. However, in the anterior maxillary region, where missing teeth might impair esthetics and satisfaction, immediate and early implant placement and loading protocols have been proposed [2]. Several reports have demonstrated that immediate implant placement in extraction sockets has comparable results with conventional placement [2,3]. Moreover, immediate implant placement was found to preserve the soft and hard tissues [4,5].

When implants are placed, adequate alveolar bone dimensions are critical for successful treatment outcomes. However, implant placement at the anterior maxillary region is faced with several anatomical limitations, including reduced buccolingual dimensions along with the presence of facial bone concavity [6,7]. Accordingly, the risk of fenestration and exposure of the implant’s apical threads might increase when implants are placed in these ridges [6,8]. Clinically, facial bone fenestration and apical threads exposure might be managed either by additional bone augmentation procedures performed to increase the buccolingual width, or by leaving it untreated [9].

Because of the mechanical interlocking relationship between the osseointegrated implants and the peri-implant bone, the alveolar bone’s geometry and local anatomy might affect the intensity and distribution of the generated stresses in the surrounding bone [10,11]. Overloading of the peri-implant bone is considered as one of the primary causes of peri-implant bone loss and implant failure [12,13].

The finite element method (FEM) has been utilized to evaluate the biomechanical aspect of oral implants by evaluating generated mechanical variables like stress and strain in the implant, bone, and prosthesis [14].

However, the biomechanical effect of exposed threads associated with fenestration defect and the consequent bone augmentation procedures has not been studied yet in relation to generated stress and strain around osseointegrated implants placed in these ridges. Therefore, this study aimed to evaluate the biomechanical impact of facial bone fenestration around implants at the anterior maxillary region using the three-dimensional finite element method.

## 2. Materials and Methods

**Figure 1:**
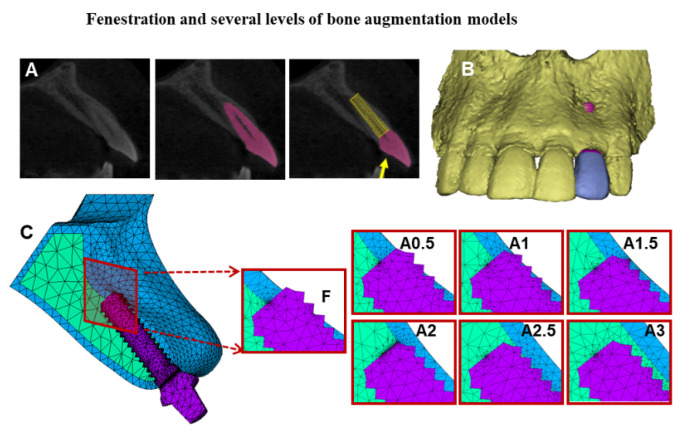
The anterior maxillary bone segment with implant and abutment models. (**A**): The sagittal section of the lateral incisor tooth and implant inserted at the same direction of the tooth root; the yellow arrow indicates the applied force direction. (**B**): The three-dimensional CBCT based model of the anterior maxillary bone segment with facial bone fenestration defect. (**C**): Meshed fenestration model and simulation of several levels of bone augmentation of the facial bone defect are displayed in the cross-sectional images (F): Fenestration model, (A0.5–A3.0): Augmentation models with 0.5 mm intervals.

A three-dimensional model of the anterior maxillary segment was constructed based on a selected CBCT image of a 34-year-old male patient with a facial concavity at the lateral incisor region. Selection was performed in two stages; in the first stage, CBCT data of patients without a gross skeletal defect, or periodontal disease, or dental malocclusion, and without any previous surgical or orthodontic treatment but with facial bone concavity were used. One CBCT data set was then randomly selected, and dicom files were imported first to Mimics 17.0 (Materialise Inc., Leuven, Belgium). Masks representing the anterior maxillary bone and teeth were generated with a threshold-based segmentation approach. The 3D geometric model was reconstructed as in Figure 1.

The lateral incisor’s root was removed to simulate extraction; however, the crown position was preserved to guide the restoration-based implant placement. Polylines function was used to generate surface lines that describe the external circumference of three sagittal sections of the lateral incisor region. The lines were used to plot points (x, y, z) coordinates. STL files, including points and lines (for lateral incisor) along with 3D model (for the anterior maxillary segment), were imported to FEA software separately (ANSYS 17.0, ANSYS Inc., Canonsburg, PA, USA). A segmented Spline Algorithm was used to recreate the outer line of the lateral incisor region. The skinning function was then utilized to create the model volumes with a cancellous core and a 1 mm thick cortical bone.

A threaded implant with a 12 mm long and 3.5 mm diameter was created and inserted following the long axis of the lateral incisor to simulate the basic fenestration model, and the defect between the implant and the extraction socket walls was filled with bone assuming full healing of the extraction socket. A straight abutment was then constructed.

Several bone coverage levels of the exposed implant were simulated by gradually shifting the deepest concavity point buccally with 0.5 mm intervals, mimicking bone augmentation surgeries with different bone fill results. The addition was performed in a curved way, using the spline function to follow the alveolar bone’s anatomy. Accordingly, seven models were constructed, as shown in Figure 1.

A tetrahedral element with 20 nodes was used for meshing the models. Elements’ numbers and meshes sizes were determined by a convergence test [15]. The models were constrained in all directions at the nodes on the distal bone surfaces and nasal crest. The implants were anchored to the bone models along the entire interface, and the same contact type was provided at the prosthesis-abutment interface. The material properties of the cancellous and cortical bones were modeled as being transversely isotropic and linearly elastic [16], which describes an anisotropic material.

A static load of 178N was applied obliquely to the crown’s long axis at 130 to the cingulum [17]. The maximum compressive stress and strain generated in the cortical and cancellous bones were calculated along with the volume of cortical bone, which exhibited compressive strain in the ranges 200–2500 µ, 2500–4000 µ, and above 4000 µ strain [18]. The maximum implant displacement was also recorded for each model.

## 3. Results

**Figure 2:**
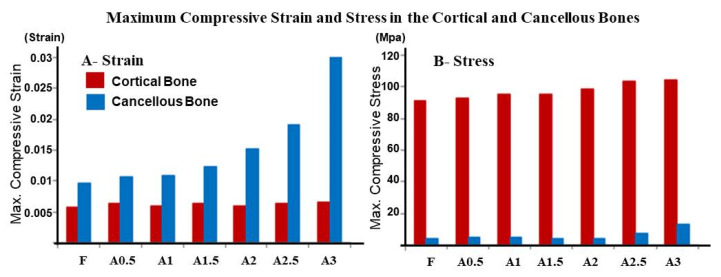
Maximum compressive strain (**A**) and stress (**B**) in the cortical (red bars) and cancellous (blue bars) bones in the fenestration (F) and augmented model (A0.5–A3).

The peak compressive stress was observed at the cervical cortical bone around the implant’s neck in all models. Compared to other augmented models, a relatively lower peak compressive stress was observed in the fenestration model with 91.3 MPa. A progressive increase in peak stress was noted as the buccolingual width increased, with the highest compressive stress value shown around the implant in the fully covered model (A3) with 104.8 MPa, as in Figure 2.

**Figure 3:**
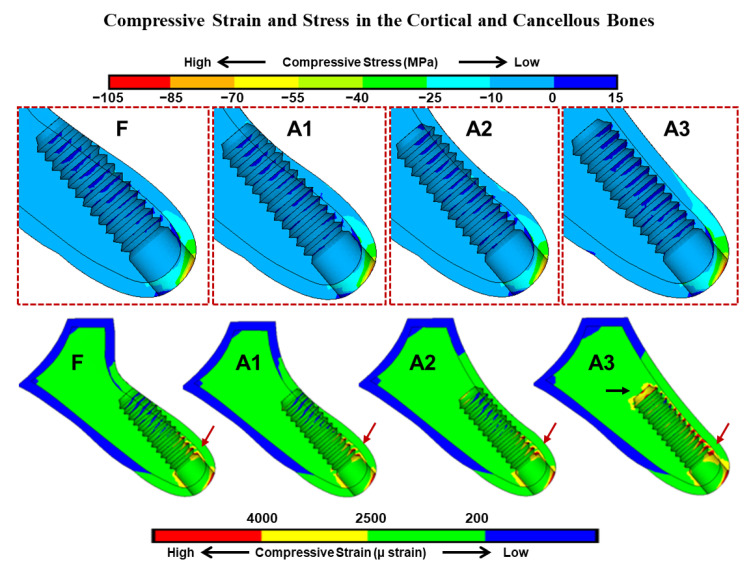
The effect of gradual augmentation of the facial bone defect and the exposure of apical threads on the compressive strain and stress generated in the cortical and cancellous bones. In the cortical bone, the maximum compressive stress and strain were shown at the facial cervical regions of all models. In cancellous bone, strain concentrations were observed at the first implant thread (red arrow), while in the A3 model, strain concentration was observed also at the implant apex (black arrow). Implant bed region in the fenestration (F) and augmented (A1–A3) models are enlarged (upper graphics). The red color contour represents the maximum strain and stress in each model.

However, in the cancellous bone, peak stress values were significantly lower than the cortical bone in all models. Maximum stress was observed at the facial side, under the cortical bone plate where a thin region is entrapped between the implant’s threads and the cortical bone in all models. On the other hand, peak compressive strain was observed at the facial aspect of the implant’s first thread in all models, with strain concentration at the implant’s apex in the A3 model (Figure 3).

The smallest implant displacement was also observed in the fenestration model with 6.3 µm. A gradual increase was then observed in implant displacement and the largest displacement of 9.1 µm was observed in the A3 model.

**Figure 4:**
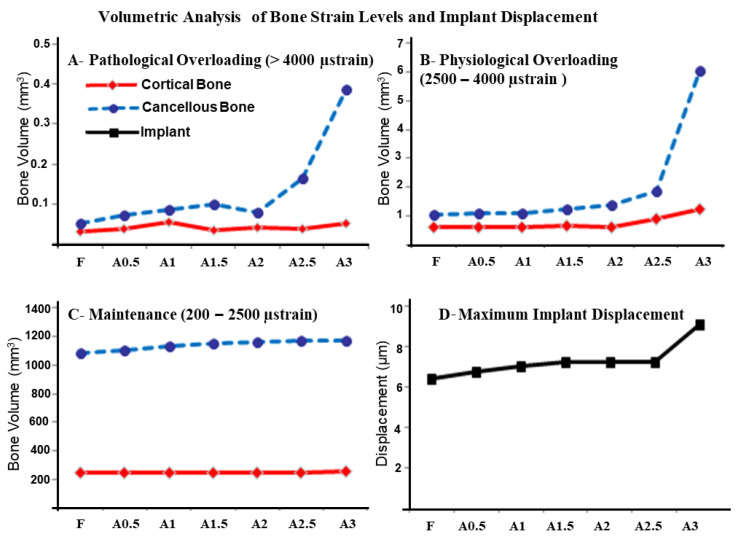
Volumetric analysis of bone strain levels and implant displacement: The volume of bone exhibited compressive strain values with (**A**)—Pathological overloading (upper left), (**B**)—Physiological overloading (upper right), and (**C**)—Maintenance (lower left) windows in the cortical and cancellous bones in the fenestration F and augmented models (A0.5–A3). The maximum implant displacement (µm) upon occlusal force application is shown in panel (**D**) (lower right).

Peri-implant bone volume exhibited compressive strain level in the range 200–2500 µ strain was relatively close but larger around the implant in the covered model. However, in the ranges 2500–4000 µ strain, it was significantly larger in above 4000 µ; however, larger bone volume exhibited a strain level over 4000 µ strain around the implant in the fenestration model (Figure 4).

## 4. Discussion

The risk of facial bone fenestration is raised in the anterior maxillary region when implants are immediately placed [8]. In this study, the biomechanical effect of facial bone fenestration and consequent augmentation procedures around osseointegrated implants has been evaluated using the FEA method.

Facial fenestration defect and level of bone fill around exposed implant’s threads influenced the maximum stress and strain results in the surrounding bone. However, upon loading the implants, maximum compressive stress was noted at the crestal cortical bone in all models (Figure 2), which was in line with most FEA studies. The mismatch between the cortical bone and implant stiffness is accountable for the concentrated stress at the first meeting point in the osseointegrated marginal bone crest [19].

Interestingly, the fenestration model showed slightly lower peak compressive stress in the peri-implant cortical and cancellous bones. However, as the buccolingual dimension at the facial concavity level increased in the A0.5 to A3 models, peak compressive stress increased gradually with maximum compressive stress generated in the A3 model. This might be attributed to the effect of bicortical fixation [20] of implants in the F-A2.5 models.

Bicortical fixation has been utilized to enhance the implant’s primary stability, especially at the posterior maxillary region [20]. However, in this study, bicorticalization resulted from the engagement of the implant’s apical threads with the inner facial cortical bone when implants were inserted following the root’s direction [8]. Accordingly, implants with exposed threads in the fenestration model might show relatively lower displacement results, which have been demonstrated in this study (Figure 4). A progressive increase in implant displacement was then noted as the cortical bone plate shifted buccally with a marked increase in implant displacement observed in the A3 model, where apical threads are entirely disengaged from the inner cortical bone, and the buccal concavity is fully augmented.

Previous FEA studies utilized the maximum compressive stress and strain as a risk scale for bone resorption, which was attributed to the accumulated microdamage that exceeds the bone’s capacity of repair [21,22]. In this study, quantitative analysis for the bone volume exhibited strain levels according to mechanostat theory has been also performed [19]. The bone volume exhibited high strain levels (>4000 µ) were very small for all models; however, the largest was observed in the A3 model, which was in line with the maximum compressive stress and strain results. On the other hand, bone volumes exhibited maintenance (200–2500 µ) and physiologic overloading (2500–4000 µ) were smaller in the fenestration model and relatively larger in the A3 model. Bone is expected to adapt to increased physiologic overloading by increased bone mineral density [21], which might improve the peri-implant bone stress [23].

Bicorticalization of implants in the F-A2.5 models might hinder the relative implant displacement, and resulted in less stress and strain being transmitted to the surrounding bone [20]. On the other hand, in the A3 model, disengagement of apical threads from the inner buccal cortical bone plate might be responsible for the favorable bone loading reflected by the larger bone volume with maintenance and physiologic overloading markedly increased but larger relative implant displacement.

As inherent assumptions and simplifications limit the direct application of quantitative results obtained from FEA studies, the aim of this study was not to predict the real in vivo stresses and strain in the peri-implant bone structures, but rather to evaluate the potential effect of fenestration defect around dental implants versus the consequent bone augmentation procedures. Assumptions usually include the model geometry, material properties, and loading scenario, among others. In this study, a single static loading scenario, as well as the bone quality, was used; however, previous studies have indicated that in comparative analysis, the relative accuracy of the results is not influenced by these parameters [23]. Moreover, the morphology of the crestal alveolar bone was assumed to be ideal with rounded representing type 3 bone [24]. In fact, crestal bone morphology was found to influence the generated stress around dental implants [11]; however, in this study, the crestal bone morphology was standardized in all models.

In implant dentistry, clinical decision making for surgical procedures might depend on both esthetics and masticatory function outcomes [25]. Likewise, avoiding complications and achieving high success rate of dental implants might rely on the biomechanical aspect as well [26]. At the anterior maxillary region, when dentists are faced with the risk of facial bone fenestration, they might proceed to bone augmentation procedures or leave it untreated. Furthermore, guided implant surgery might be suggested here for accurate implant placement [27]. From a biomechanical point of view, fenestration around dental implants at the maxillary anterior region does not contribute to the overloading of the surrounding bone. This might support the clinical reports that questioned the necessity of intervention to cover the facial fenestration defects around dental implants [9]. On the other hand, further clinical studies with extended follow-up periods might be encouraged to evaluate the long-term effect of fenestration around dental implants.

## 5. Conclusions

Within the limitations of this study, facial bone fenestration defects around dental implants and bone augmentation procedures might influence the generated peri-implant compressive stress and strain; however, exposure of the implant’s apical threads does not contribute to the overloading of surrounding bone structures. On the other hand, increasing the buccolingual width and eliminating the facial concavity might favorably increase the peri-implant bone volume exhibited physiologic overloading strain levels.

## Data Availability

CBCT data set, STL files and FEA models used and constructed in this study all are available upon reasonable request from the corresponding author.
